# Parliament and the Professions

**Published:** 1921-05-21

**Authors:** 


### Parliament and the Professions.

### Pasteur Treatment.

The Minister of Health was asked how many persons bitten by alleged or suspected mad dogs since the first case of rabies was notified in this country after the war have refused Pasteur treatment, and if any of these have contracted hydrophobia. Sir Alfred Mond said he was aware of only two persons, bitten by dogs which were reasonably suspected to be rabid, who refused Pasteur treatment. Neither developed hydrophobia.

### West Middlesex Hospital.

Mr. D. Herbert asked the Minister of Health if his attention has been called to the alleged unfair treatment of probationer nurses at the West Middlesex Hospital under the Brentford Board of Guardians; if he is aware of the serious discontent thus caused amongst the nursing staff; and what steps he proposes to take in the matter. Sir Alfred Mond replied that he had been in communication both with the Board of Guardians and with the various associations which have taken the matter up on behalf of the nursing staff. He had offered, if they so desire, to arrange a meeting between these associations and officers of the Ministry, at which the case can be further considered in the light of the explanation which he had received from the Guardians.

### Maternity Cases in Ireland.

Viscountess Astor inquired of the Chief Secretary for Ireland as to possible hardship, in view of police and military restrictions, in cases of child-birth. Mr. Henry replied that all doctors and nurses who choose to apply for curfew permits can obtain them. Doctors and nurses are also expressly exempted from motor restrictions and can use motor vehicles irrespective of distance or time.

